# The London and Counties Medical Protection Society (Limited)

**Published:** 1893-08-26

**Authors:** 


### The London and Counties Medical Protection Society (Limited).

A warrant has been issued for the apprehension of an Indian oculist, styling himself Kream Bocesh, on a charge preferred against him by the London and Counties Medical Protection So ciety of maliciously and unlawfully cutting and wounding a patient. It is earnestly requested that information as to any Indian oculists at present practising shall be at once forwarded to the Hon. Secretary of the Society, Dr. Hugh Woods, 11, Archway Road, Highgate N.

The following interesting letter on "Electric Spectacles," published in the *Standard* of 23rd inst., is an apt commentary on the foregoing paragraph. We reproduce it in the hope that all sufferers who have a knowledge of similar cases of gross fraud will at once communicate with Dr. Hugh Woods, the able Honorary Secretary of the Medical Protection Society.

"*A propos* of the sale of the above spectacles, it may interest your readers to learn that this industy is not confined to the North.

"A few days ago a poor woman presented herself at ——— Hospital to have her eyes examined for glasses. Seeing that she held a pair in her hand—a very shoddy pair, worth less than a shilling—I asked where she had obtained them. She informed me they were electric glasses, and, in answer to inquiries as to who sold them to her, said, 'A gentleman called to see me and told me my eyes were very bad, and needed glasses.' She was very subject to rheumatism, so he represented to her that they would cure her eyes, and also her rheumatism. As a great favour he let her have them for a guinea. The patient was an old widow, who lived in an almshouse, and the glasses were quite useless to her.

"Perhaps you will be good enough to allow me to refer to some other cases in which the poor have been mercilessly fleeced. One case is that of a poor woman whose husband was a carpenter confined in a lunatic asylum. She brought her little girl with an incurable eye affection—the front of the eye was destroyed. It was clear from the recent and numerous scars on the temple that she had been in the hands of an impostor, so I asked to whom she had been. Her replies confirmed my convictions, and it transpired she had paid him thirty pounds 'He said he could cure her, and I sold my furniture and borrowed of anybody who would lend me anything, and begged from anybody who would give me anything.'

"Another case is that of a poor woman who 'had a cataract removed" by one of these gentlemen. She said she had had a cataract removed from the right eye. Despite what she said, there was the cataract. I asked her if she saw it after its removal. Her reply was in the affirmative; that the doctor spread it out on the back of his hand afterwards to show her, and that it was a thin white skin. A cataract is the shape of a bi-convex lens, and is about a quarter of an inch thick. A week or two later the cataract was removed in the hospital. This poor woman paid twenty pounds for a bogus operation, and gave a testimonial testifying to his great skill, which I believe was attested by two witnesses.

"These cases will suffice to show the rascality that is practised on poor poople. One word as to the procedure followed by these high-minded gentlemen. They affect to cure everything—the only thing they ask is time, and a case pronounced incurable by the ablest men is what they like best. The visits paid depend upon the pocket and obvious credulity of the individual. Whilst there is enough left to pay a fee (five shillings to four guineas) eventual cure is certain; but when the victim cannot pay the case is very trying; still, he could cure them if they could attend long enough. Instances in which incurable cases are mulcted of their last penny simply abound, and could their victims see to read this letter I have no doubt they could furnish your readers with tales that are almost incredible.

"The law as it stands is no protection to those who need it. A man who has spent his last penny, or even one who has a pound or two left, is scarcely the person to have the courage to face its annoyances and uncertainties. The remedy is very simple—let all such occupations as that of an oculist be absolutely restricted to those whose diplomas are a guarantee of efficiency and good faith."

